# Providers’ perspectives on the reproductive decision-making of *BRCA*-positive women

**DOI:** 10.1186/s12905-022-02093-2

**Published:** 2022-12-08

**Authors:** E. S. Dason, L. Drost, E. M. Greenblatt, A. S. Scheer, J. Han, M. Sobel, L. Allen, M. Jacobson, T. Doshi, E. Wolff, E. McMahon, C. A. Jones

**Affiliations:** 1 Department of Obstetrics and Gynecology, Temerty Faculty of Medicine, University of Toronto, Toronto, ON M5G 1E2 Canada; 2 Department of Obstetrics and Gynecology, Mount Sinai Hospital, Sinai Health System, Toronto, ON M5G 1X5 Canada; 3 Department of General Surgery, St. Michaels Hospital, Unity Health Network, Toronto, ON M5B 1W8 Canada; 4 Department of Obstetrics and Gynecology, Women’s College Hospital, Toronto, ON M5S 1B2 Canada; 5 Bloomberg Faculty of Nursing, University of Toronto, Toronto, ON M5T 1P8 Canada; 6 Mount Sinai Fertility, 7th Floor, 250 Dundas St. W, Toronto, ON M5T 2Z5 Canada

**Keywords:** Reproductive decision-making, Decision support, Cryopreservation, Gene mutations, Qualitative research

## Abstract

**Background:**

Reproductive decision-making is difficult for BRCA-positive women. Our objective was to assess the complexities of decision-making and identify decisional supports for patients and providers when discussing reproductive options prior to risk-reducing salpingo-oophorectomy (RRSO).

**Methods:**

This study was of qualitive design, using data collection via semi-structured interviews conducted from November 2018 to October 2020. Individuals were included if they were identified to provide care to *BRCA*-positive women. In total, 19 providers were approached and 15 consented to participate. Providers were recruited from three clinics in Toronto, Ontario located at academic centers: [1] A familial ovarian cancer clinic, [2] A familial breast cancer clinic and [3] A fertility clinic, all of which treat carriers of the *BRCA1*/*BRCA*2 genetic mutation. The interview guide was developed according to the Ottawa Decision Support Framework and included questions regarding reproductive options available to patients, factors that impact the decision-making process and the role of decisional support. Interviews were transcribed and transcripts were analyzed thematically using NVIVO 12.

**Results:**

Providers identified three major decisions that reproductive-aged women face when a BRCA mutation is discovered: [1] “Do I want children?”; [2] “Do I want to take the chance of passing on this the mutation?”; and [3] “Do I want to carry a child?” Inherent decision challenges that are faced by both providers and patients included difficult decision type, competing options, scientifically uncertain outcomes, and challenging decision timing. Modifiable decisional needs included: inadequate knowledge, unrealistic expectations, unclear values and inadequate support or resources. Identified clinical gaps included counselling time constraints, lack of reliable sources of background information for patients or providers and need for time-sensitive, geographically accessible, and centralized care.

**Conclusion:**

Our study identified a need for a patient information resource that can be immediately provided to patients who carry a *BRCA* genetic mutation. Other suggestions for clinical practice include more time during consultation appointments, adequate follow-up, value-centric counseling, access to psychosocial support, and a specialized decisional coach.

**Supplementary Information:**

The online version contains supplementary material available at 10.1186/s12905-022-02093-2.

## Introduction

Women carriers of a *BRCA1* or *BRCA2* disease-causing genetic variants are predisposed to the development of hereditary breast and ovarian cancer (HBOC) [1]. Depending on the *BRCA* mutation, affected women will have up to an 87% risk of developing breast cancer and a 54% risk of developing ovarian cancer by the age of 70 with a median age of diagnosis of 44–48 for breast cancer and 54–60 for ovarian cancer [1–3]. Thus, BRCA-carriers are faced with uncertainty regarding when they may face a cancer diagnosis causing significant distress [4]. Patients are typically offered several preventative surgeries (mastectomy, risk-reducing salpingo-oopherectomy) and rigorous routine screening options [5]. One such option, risk-reducing salpingo-oophorectomy (RRSO), is offered at the age of 35 for *BRCA1* carriers and at the age of 40 for *BRCA2* carriers [4, 6, 7]. The decision to undergo RRSO involves many considerations such as surgical menopause, change in sexuality and body image, and fertility loss [4, 6]. At the time of *BRCA* gene discovery, approximately 40% of women are actively engaged in family planning [8].

As BRCA mutations are passed on to 50% of offspring, BRCA-carriers have a number of reproductive considerations. Family planning options include fertility preservation in the form of oocyte cryopreservation or in vitro fertilization prior to RRSO, spontaneous conception in a shortened timeline prior to cancer diagnosis or RRSO, and the use of pre-implantation genetic testing to prevent passing on the mutation [9]. Prior research has demonstrated that this is a difficult decision for patients for a multitude of reasons, including the involvement of their partner in decision-making, the uncertainty surrounding their cancer status, a shortened reproductive timeline and the consideration of gene inheritance [10]. Family planning is a deeply personal choice; however, patients often lack the medical expertise to make decisions in the face of underlying medical concerns and thus patients have expressed the need for decision-making support [11].

In a shared decision-making model, the provider shares information and the patient shares their values to come to a joint patient-led decision [12]. This model has been shown to improve decision-making in obstetrics and gynecology [12]. Although studies have focused on factors influencing female *BRCA*-carrier’s reproductive decision-making, no studies have addressed shared-decision making between provider and patient [1, 2, 4, 13, 14]. Providers are an important population to assess as they see large numbers of patients and thus have participated in these discussions before. They must have the medical expertise to guide patients through their decision, but they must also understand a patient’s values in order to appropriately frame the decision according to what the patient needs. Given that the existing data comes from a patient’s perspective, it is important to understand the provider’s perspective to appropriately address and improve decisional quality. The Ottawa Decision Support Framework (ODSF) is one tool that offers a standardized approach to assess a populations’ decisional needs [15].

This study aimed to assess decision-making needs of patients and providers from the provider’s perspective when discussing reproductive options with female *BRCA* genetic mutation carriers prior to RRSO.

## Materials and methods

This study was of qualitative design using data collection via semi-structured interviews conducted from November 2018 to October 2020.

### Recruitment

The following populations were selected through purposive and convenience sampling: [1] Healthcare providers (gynecologists, nurses) at the Familial Ovarian Cancer Clinic (FOCC) at Women’s College Hospital, [2] Healthcare providers (reproductive endocrinology and infertility (REI) physicians, REI fellows, nurse practitioner, genetic counselor) at Mount Sinai Fertility (MSF) and [3] Healthcare providers (genetic counselor) at the Familial Breast Cancer Clinic (FBCC), Mount Sinai Hospital, Toronto, Ontario. Providers were recruited via email invitation and consent was completed at the time of in-person or telephone interview.

The FOCC is a highly specialized clinic that focuses on RRSO and post-operative care following RRSO for BRCA-carriers. MSF is an academic fertility clinic that receives direct referrals from FOCC. The FBCC is a highly specialized clinic facilitated by genetic counselors for individuals with a personal and/or family history of breast cancer suspicious for a hereditary predisposition and is often the first point of diagnosis for patients carrying the *BRCA* mutation.

### Data collection

Data was collected through semi-structured telephone (9) or in-person (6) interviews from November 2018 to October 2020 based on an interview guide adapted according to ODSF principles [16]. Interviews lasted 15–30 min. Interviews were audio-recorded and transcribed verbatim by a study investigator (E.S.D., L.D., T.D.). Transcriptions were checked for accuracy by a second study investigator (E.S.D., L.D., T.D.). Concepts of qualitative research such as reflexivity of the study investigator, emergent findings, and ongoing analysis were all integrated into data collection and analysis. Sampling was emergent and guided by thematic saturation [17].

### Study investigators

Interviews were conducted by the investigators E.S.D., L.D., E.W. and J.H as well as by the research assistant, M.S. Investigators (C.J., M.L.S., M.J., L.A., M.B., E.G.) who were involved in direct patient care were not involved in conducting interviews or initial coding. They were involved in data analysis and interpretation of anonymized results.

### Interview guides

An interview guide, adapted from the ODSF, (Additional file 1: Appendix 1) consisted of a mix of open-ended and closed questions about the options available to patients, factors that impact the decision-making process and the role of decisional support [16, 18]. The interview guide was considered fluid and interviews were explored according to concepts brought up by participants. Interviews in the study informed iterations of the interview guide in an effort to explore concepts more fully.

### Data analysis

Data analysis was guided by the concepts of qualitative description and thematic analysis to identify users’ needs and involved the development of codes, concepts, categories, themes and theories [17, 19, 20]. Codes were informed by the ODSF [16]. Interviews were independently coded using NVIVO 12 by two study investigators, L.D. and S.D. An audit trail of memos and coding was kept ensuring reliability of data. Similarities and discrepancies in coding, concepts and themes were then discussed by L.D., S.D., C.J., E.G. and T.D. to come to a conclusion.

### Ethics approval

Approval from the Mount Sinai Hospital Research Ethics Board (# 18-0086-E) and Women’s College Hospital Research Ethics Board (# 2018-0050-E) was obtained prior to initiation of the study.

## Results

### Provider demographics

Nineteen providers were approached for participation via email with 15 providers consenting to participation; four providers did not respond to recruitment emails and did not provide a reason for non-response. Most providers were female [13/15]. Providers included REI physicians [4/15], REI fellows [3/15], general OB/GYNs [3/15], a gynecological oncologist [1/15], a nurse practitioner specializing in fertility preservation (1/15), a registered nurse at the FOCC [1/15], a genetic counselor specializing in PGT (pre-implantation genetic testing) [1/15], and a genetic counselor at FBCC specializing in *BRCA* genetic testing [1/15]. Providers had varying years of experience, with 7/15 having fewer than 10 years, and 8/15 having over 10 years.

#### ODSF decisional needs concepts

The ODSF was used as a framework to outline decisional difficulty according to provider views. ODSF concepts that were predominant included decisional difficulties inherent to the decision (i.e., difficult to change), modifiable decisional needs, decisional conflict and inadequate support and resources. Potential decisional supports were identified. A decisional need is defined as a “deficit that can adversely affects the quality of a decision and requires tailored decision support.”^13^ Decisional conflict arises from both the inherent nature of the difficult decision and modifiable decisional needs and leads to voiced uncertainty regarding the decision-making process.^13^

#### Decisional difficulty inherent to the decision

Reproductive decision-making for *BRCA*-carriers is difficult for a multitude of reasons; some decisional deficits can be modified, and others are inherent to the decision itself. These are discussed separately below.

The inherent decisional elements that made decision-making difficult for BRCA-carriers and their providers included the number of reproductive decisions, the number of reproductive options available to patients, the timing of the decision, and the uncertainty surrounding these decisions.

The three main reproductive decisions that all providers spent time discussing with female BRCA-mutation carriers are outlined below.

**“Do I want children?”** – This decision, for some patients, may be, “Do I want to have more children?” All providers shared that this first decision requires significant patient motivation to make at a time in the patient’s life when this may not be at the forefront of their initial life plan. At the time of BRCA discovery, many patients chose to defer this decision. Deferral of this decision, or not taking action to freeze eggs or embryos at a time when patients are healthy and cancer-free was a factor associated with regret. Fertility providers specifically encouraged patients to ask themselves, “Am I comfortable with the possibility of never having children?” Providers who saw patients earlier in their lives (i.e., genetic counselors at the FBCC clinic or gynecologists at the FOCC clinic) were less likely to explore this question in detail with patients. Patient motivation to have children was a major factor identified that influenced ongoing counselling.

**“Do I want to take the chance of passing on this mutation?” –** One genetic counselor involved in initial diagnosis of *BRCA* shared that most patients do not consider this decision at the forefront of their family planning. In contrast, fertility providers shared that this was an important decision made by patients who were motivated to pursue fertility consultation. A genetic counselor involved in PGT counseling commented that this raises an important ethical dilemma for patients which they often struggle with. If patients were very clear that they did not want to pass on the mutation, then this required the patient to take active steps to avoid natural conception. It led to another question, “Do I want to have a genetically linked child?” Further options then included assisted reproductive technology (ART) options, PGT and consideration of adoption. If patients were unsure about their answer to this question, they still needed to consider taking steps to prevent natural conception while they took the time to decide.

**“Do I want to carry a child?”** – FOCC providers shared that patients were often concerned about the effects of endogenous and exogenous hormones on their risk of breast cancer, especially in the context of pregnancy. Alternative options for patients might include ART with subsequent surrogacy, and adoption.

Other important decisions that provide more context for reproductive decision-making highlighted by providers include decisions on treatment and prevention of breast and ovarian cancer. Some of these decisions included whether to pursue prophylactic mastectomy versus enhanced screening, whether to undergo RRSO, and whether to take hormone therapy after RRSO. Deliberating between reproductive options required assessing and weighing personal patient considerations that contribute to establishing their personal value systems. Providers were largely unsure how patients deliberated amongst their considerations, determined their values and how they arrived at a decision.

“They were so overwhelmed with having to deal with both issues together like do I do the surgery to prolong my life and then if I'm prolonging my life, I have another not a smooth road to having children.” *Provider 8, FOCC RN*

Patients’ answers to these three major questions defined the scope of reproductive options available to them. These options and the specific considerations of each option identified by providers are outlined in Table 1. While fertility providers were well-versed in available options, other providers indicated they were unsure about what could be offered. All three decisions were ultimately difficult for patients to make due to multiple competing options and scientifically uncertain outcomes. PGT was an added layer of complexity for patients as it required more specific genetic counselling that could not often be provided by a fertility physician alone.

**Table 1:** Reproductive options for people carrying BRCA mutation and selected patient considerations

Reproductive option	Identified considerations
Natural conception + / − prenatal diagnosis	Genetic link to children Possibility of child carrying *BRCA* Prenatal diagnosis is possible Potential risk of carrying pregnancy with underlying breast/ovarian cancer risk (though not supported by current evidence) Age-related fertility decline
Assisted conception with cycle monitoring + / − intrauterine insemination + / − ovarian hyperstimulation + / − prenatal diagnosis	Genetic link to children Possibility of child carrying *BRCA* Prenatal diagnosis is possible Potential risk of carrying pregnancy with underlying breast/ovarian cancer risk (though not supported by current evidence) Risk of intervention
Egg freezing	Genetic link to children Reproductive autonomy maintained allowing conception with a partner or donor sperm in the future Halts age-related fertility decline Allows possibility of PGT in the future or surrogacy in the future Delays reproductive decision-making Risk of intervention Risk of attrition
Embryo freezing + / − donor sperm + / − pre-implantation genetic testing for aneuploidy and/or *BRCA* + / − surrogacy	Genetic link to children Halts age-related fertility decline Can screen for BRCA mutations in embryos Can use donor sperm or partner sperm Risk of intervention Risk of attrition
Egg donation + / − donor sperm	No Genetic link to children Removes risk of passing on *BRCA* mutation to child
Surrogacy	Removes potential risk of carrying a pregnancy Allows genetic link to children
Adoption	No Genetic link to children Removes risk of passing on *BRCA* mutation to child Removes potential risk of carrying a pregnancy
Child-free living	Removes risk of passing on *BRCA* mutation to child Removes risk of carrying a pregnancy

The timing of making a reproductive decision was felt to be difficult for patients by all providers. There was a perceived urgency due to a shortened reproductive timeline and the constant risk of impending cancer. FOCC providers focused on this decision in proximity to the recommendation for RRSO.

“I mean the problem is that it's a multifactorial process and the first decision that they have to make, if they come to see us, is the timing of risk reducing surgery.” *Provider 4, General OBGYN*

In addition, many women are found to be at an unreceptive decisional stage where they aren’t truly considering reproduction at the time of *BRCA* gene discovery.

“For patients who are doing it because they feel that they need to have their preventive surgery, but they're not done with their families yet, they have a lot of difficulty in the decision.” *Provider 6, general OBGYN*

Similarly, all providers identified that the impending cancer treatment contributed to sense of finality to reproductive decision-making.

“I think that knowing that they can't really go back on their decision in the future -if they decide to go ahead and remove their ovaries. The decision that they will have made, they'll have to stick with and if they made a mistake, then their only option for parenthood in the future would be either egg donation or adoption.” *Provider 9, REI fellow*

Importantly, there was significant uncertainty shared by providers that impacted their reproductive discussions with patients. Providers shared that patients felt uncertain of the associated physical and emotional risks of ART and expressed uncertainty regarding the scientifically uncertain outcomes. Fertility providers felt constrained by the lack of available data to provide to patients regarding ART outcomes, especially live birth rate in the context of oocyte cryopreservation.

“'Do I think I'll ever use these eggs?' Some of them aren't sure …. so that makes it hard for people because I’m not going to put myself through potentially risky procedure even though the risk is quite low…And you can't guarantee that there will be live birth from these eggs.” *Provider 1, nurse practitioner specializing in fertility preservation*

All providers expressed that the presence of voiced patient uncertainty surrounding the potential change in family planning goals throughout life, uncertainty around future fertility potential, uncertainty about the impact of the treatment on cancer status and uncertainty of the technology involved in ART.

“Especially for women who are single, I think their ideal choice would be [to] just to be able to forget about all this, and to meet sort of the person they want to be their life partner, and to be able to have children when they’re ready to have children and I think they feel like you know they have to make these decisions which are not easy decisions at a time when they really don’t know where their life might take them.” *Provider 13, REI physician*

Providers shared that this uncertainty often led their patients to feel scared, anxious, depressed, and overwhelmed. Some providers shared that patients who made this decision voiced that they felt a sense of control amongst the myriad of unknowns, although patients continued to question their choice.

“I just find that the patients feel very conflicted…internally. Like they see the upsides of doing it. And they also are hesitant about like everything that's involved and if they really want to pursue it.” *Provider 3, REI physician*

#### Modifiable decisional needs

There were several identified decisional deficits that are considered modifiable. All providers felt that patients generally had a lack of knowledge about reproductive options, although fertility providers felt they were well-equipped to address this lack of knowledge directly.

“So, I usually find that when I bring this up in the clinic, people are like ‘oh my goodness, what are you even talking about?’ There’s mass confusion over egg versus embryo freezing. And people don’t understand the idea of committing the parents of the genes of the pregnancy. They think that they can freeze embryos and then later on go and decide who the partner is going to be.” *Provider 7, general OBGYN*

FOCC and FBCC providers also felt that they lacked knowledge about the reproductive options available and were uncomfortable participating in fertility counselling.

“Probably at some level, I think at some level there is a real lack of knowledge. And whenever there's lack of knowledge, some people they just kind of gloss over it. I don't think even us as practitioners know all of the data and all of the information around what it costs, what are the different options, how you could do this.” *Provider 5, Gynecologic Oncologist*

Similarly, fertility providers also felt that patients received conflicting information from other healthcare providers.

“They may be getting…mixed messages from different specialists who aren't as familiar with like how quick and how spaced we can usually do this.” *Provider 3, REI physician*

Fertility providers felt that patients had unrealistic expectations of ART outcomes and struggled with providing a false sense of hope.

“There’s also the emotional risk if IVF doesn’t translate to success in the future, so the idea that you’ve frozen eggs or-autologously, eggs or embryos, and then you have high expectations for this batch in the future, but you might…thaw in the future, transfer, no baby.” *Provider 10, REI physician*

All providers felt that patients largely had inadequate emotional support.

“We don’t have access to [a social worker] through our clinic, just because we don’t have the resources, it’s a small part of what we do. But I assume that they have those resources through the FOCC, and if they don’t, they certainly should. Because all the women there are in a situation in some way or another.” *Provider 13, REI physician*

All providers felt that health information was largely siloed, and patients lacked access to expertise in reproduction.

“Well, I guess it’s always good to get all the players involved… we do still function a little bit in silos, right? I think it would probably be good if we were all working from the same playbook to a certain extent.” *Provider 13, REI physician*

Most providers shared that their patients usually accept the complex medical information from the various healthcare practitioners involved in their care but then lack support in deliberating amongst their options and making a decision.

“They take our information. They take written material about what IVF is. And sadly - and then they talk to their oncologist or their general gynecologist, whoever's doing the risk-reducing surgery. And unfortunately, that's it. And I think there's a real gap in helping them make the decision.” *Provider 10, REI*

Possible areas for improved support are explored below.

### Availability and provision of background information

Most providers acknowledged that background information about the available fertility options and associated considerations should be provided to patients soon after the diagnosis of carrying a *BRCA* mutation; this was felt to be lacking in the current system. Sources of background information, especially through reliable sources that employ multimedia formats (i.e., videos, phone applications, website access, written information) to appeal to different people with different learning needs, was felt to be lacking and important for this population. FOCC providers and FBCC providers also felt that this background information on the available reproductive options would be useful for themselves to understand how to counsel patients.

### Challenges in current clinical structure

Providers also struggled with limited interactions with patients. They felt that they did not have time to get to know the patient well enough to help guide them through their values or belief systems. Providers shared that building a rapport was important to engaging in these discussions about reproductive decision-making. Similarly, all providers felt constrained by the static nature of their relationship with the patient.

“We, in our time meeting with patients, see them only in a static moment in their life and don’t always have the opportunity to follow up with them and know how things might change for them with time, with age, as they may be contemplating fertility and having children.” *Provider 15, genetic counselor at FBCC specializing in BRCA genetic testing*

Fertility and general OBGYN providers indicated that often there was a lack of time available to spend counselling these patients due to the inherent complexity of their decisions and options. Providers ultimately felt that having more time during consultations or follow-up appointments would better help them to share in decision-making with patients.

“I just wish we had a little bit more time, also, to talk to them about all these different options because it is complicated, and it can be very overwhelming for a lot of them.” *Provider 11, REI fellow*

In addition, centralization of care was felt to be important, and this was expressed in different ways. For example, all healthcare professionals involved in counselling patients (i.e., breast oncologists, gynecological oncologists, genetic counselors, gynecologists) would ideally be relying on the same source of information to avoid misinformation and enhance consistency in counselling.

Integration of psychosocial support into current care models was highlighted, both because the decisions themselves were emotionally confronting and the options to choose from carried an emotional burden. Providers were unsure about where patients receive support for this significant emotional burden. While FOCC providers thought this support was available through the fertility clinic and were willing to refer to the program social worker, fertility providers thought this support was available through FOCC.

Lastly, providers indicated that patients should have timely access to appropriate professionals (fertility providers, genetic counselors) with geographical access considered as well (i.e., virtual counselling).

### Perceived decisional roles

FOCC providers and fertility providers participated in two different shared decisions with patients. The first decision, which was facilitated by the FOCC provider, involved whether a patient needed to see a fertility provider, while the second decision, which was facilitated by a fertility provider, involved deciding amongst the reproductive options.

Perceptions of their decisional roles varied amongst providers. All providers acknowledged that patients need to prioritize their life values and both decisions were ultimately patient-led, but some providers felt comfortable sharing the decision with the patient while others felt their role was more in the provision of information alone. Some providers shared that they felt their role in decision-making was limited to just sharing the medical information and the decision-making amongst the options occurred primarily outside of their consultation.

“So, my role is to introduce the ideas to the people and so that they start thinking about them. I don’t think that we really try to sway people in either direction, we really just say ‘this is what’s out there and you should know what’s out there.” *Provider 7, General OBGYN*

Several providers also took a more active role by encouraging patients to verbalize their considerations and then discussing them. These providers then used the patient considerations to help patients deliberate amongst their options.

“I think usually it's more about providing information, providing the support, and then talking them through all the different eventualities. Like if this, then this, how would you feel about that et cetera and just being available to answer any questions that they have.” *Provider 11, REI fellow*

FOCC providers felt that their own lack of knowledge of the available fertility options made it difficult for them to initiate a conversation about reproductive options with patients. They were more comfortable with referral to a fertility provider to discuss these concerns. However, providers acknowledged that a challenge is that there was often a wait time before fertility consultation that led to more patient anxiety.

“Sometimes if we see them and [previous providers said] yeah, they can see you in two months. And then during that time they can spiral in terms…not understanding the options and fearing the worst.” *Provider 12, REI fellow*

### Peer support

Participants felt that discussion groups of patients facing the same decision or who had already gone through the decision that were moderated by healthcare professionals would be beneficial. Providers commented that patients often sought how other people had made the same decision they were facing.

“People do like to know ‘what have most people in my situation done?’ How many people have come back to use what they’ve frozen, and how successful were they?’ We don’t have the ability to connect them with other peers who’ve done this.” *Provider 1, nurse practitioner specializing in fertility preservation*

## Discussion

This novel study is the first to analyze the provider views on difficulties supporting reproductive decision-making for *BRCA*-positive women prior to RRSO. Our results demonstrate that this is a difficult decision with inherent decisional difficulties (i.e. multiple options, shortened reproductive timeline), modifiable decisional difficulties (i.e. lack of awareness of fertility options) and possible decisional support mechanisms (i.e. provision of background information) were clearly demonstrated through the participant responses [16]. Our study further highlights prior research calling for more support for reproductive-decision making amongst *BRCA-*carriers as this is a difficult decision.

Prior studies have identified the complexities involved in this decision for patients including the impact of personal and family history of cancer, concern about passing on the gene, the tension between cancer prevention and family planning, the number of family planning options and a shortened reproductive timeline [2, 5, 6, 11, 21]. Importantly, our findings echoed prior research and thus highlighted that providers are well-aware of patient difficulties with decision-making. In an effort to improve upon shared decision-making as a whole, this study further adds provider difficulties in decision-making. One such difficulty is that most non-fertility providers were not well-versed in the available fertility options which limited their ability to participate in fulsome discussions and contributed to conflicting shared information with patients.

It has been shown previously that healthcare providers assume that patients make rational decisions on treatment and prevention strategies after discovering their HBOC risk [22]. However, studies have shown that patients do not solely rely on medical evidence and available probabilities of risk, instead, they reframe the decision according to their own personal values, family cancer experiences and influences from their social support networks [5, 22]. In addition, patients carrying the *BRCA* genetic mutation have previously indicated that fertility options were not well discussed by healthcare providers and expressed a desire for further support in decision-making [2, 23]. In our study, providers recognized the influences of a patient’s personal belief system. Providers felt that patients were not getting adequate support to incorporate the different beliefs, viewpoints, and assumptions into their reproductive choice. However, there were differing perceptions of the role of a healthcare provider. Most healthcare providers do not feel they should share in decision-making and provide decision support. They view their role as only sharing medical information. This further highlights an existing gap in decision-making support for patients who are facing this choice. As other studies have focused on the decisional needs of patients, it is clear from our results that healthcare providers also need more decisional support to share in patient-led decision-making.

This study had limitations. Firstly, the views expressed may not necessarily be representative of the wider population as only providers at one academic fertility clinic and two highly specialized clinics that serve only patients with familial breast and ovarian cancer were included. Every potential provider view (i.e., family physicians, breast oncologists) on reproductive decision-making in this population was not captured. Strengths of this study include the fact that participants were drawn from clinics in a tertiary care center that are highly specialized in the care of BRCA-positive women and therefore see large numbers of patients.

Importantly, this study identified that this is a highly complex decision for both patients and providers. This was primarily because of the number of decisions that patients face and the options that were available for each decision depending on a patient’s answer. We clearly identified the need for two different forms of decisional support for these patients. First, the provision of background information for both non-fertility providers and patients is necessary. Second, a consultation with a healthcare provider well-versed in available reproductive options or a specific decisional coach to address all decisional needs adequately and help patients deliberate amongst the options according to their personal values is needed.

Based on this data, our research team has undertaken the development of a simplified background information handout for patients and non-fertility providers who counsel patients with *BRCA* mutations (Additional file 2: Appendix 2). The information emphasizes the impact of both the *BRCA* mutation and RRSO on fertility and the available reproductive options and includes visual formats of probable outcomes. This handout would also serve to provide the same information for patients across different clinics, as an effort to standardize the reproductive information they receive. This handout was developed based on frequently asked questions by patients with input from fertility providers, providers who care for *BRCA* patients (general OBGYNs, genetic counselors), patients and a communication specialist. It was adapted to a grade 9 readability level.

Suggestions for clinical practice include raising reproductive choices early for BRCA-positive patients (i.e. introduction of the topic at the time of discovery of BRCA), increased clinical time allocation, review of specific patient values, ensured follow-up and access to appropriate psychosocial support. Prior studies demonstrate that ongoing support for patients with genetic mutations who are making decisions in the face of fluctuating life circumstances aids patients, and therefore should be integrated into the structure of a specialized clinic for patients with the *BRCA* mutation [13, 16]. Increased clinical time allocation may involve a pre-consultation introduction to information (i.e. direction to a website, provision of an information sheet and/or specific webinars) with a subsequent meeting with a physician comfortable in discussing reproductive options with patients. In addition, follow-up in 3–6 months should be arranged to allow patients to consider their options and their own personal values. At this follow-up, space should be allowed for the patient to explore their value systems in the context of their reproductive options and raise appropriate questions. This would also allow providers to build rapport with patients, a concern identified in this study. Another option for decision support that would address these provider concerns about participating in this type of shared decision-making might be clinical personnel identified as a specialized decisional coach (i.e., RN, patient navigator), especially within specialized clinics for *BRCA*-positive women. This coach would ideally understand the background information of the decision and be able to help a patient with their personalized, nuanced deliberations. Suggestions for how a decisional coach should be trained are indicated in the ODSF [15]. Lastly, identification of resources for psychosocial support for these patients should be prioritized. Whether the interventions subsequently improve decisional outcomes would need to be assessed in future studies.

## Conclusion

In summary, decisional support is needed to address the modifiable decisional needs for both patients who carry a *BRCA* gene mutation and providers in reproductive decision-making prior to RRSO. In response to the findings in this study, we have created a patient information handout and identified implementable clinical practice changes to support these patients and providers in shared reproductive decision-making.

## Supplementary Information

**Additional file 1:** This was the semi-structured interview guide used by the authors.

**Additional file 2:** This is a patient hand-out that can be used by both non-fertility providers and patients.

## Data Availability

The datasets generated and/or analyzed during the current study are available from the corresponding author on reasonable request.

## References

[1] Werner-Lin A (2008). Beating the biological clock: the compressed family life cycle of young women with BRCA gene alterations. Soc Work Health Care.

[2] Quinn GP, Vadaparampil ST, Tollin S, Miree CA, Murphy D, Bower B (2010). BRCA carriers’ thoughts on risk management in relation to preimplantation genetic diagnosis and childbearing: when too many choices are just as difficult as none. Fertil Steril.

[3] Kuchenbaecker KB, Hopper JL, Barnes DR, Phillips KA, Mooij TM, Roos-Blom MJ (2017). Risks of breast, ovarian, and contralateral breast cancer for *BRCA1* and *BRCA2* mutation carriers. JAMA.

[4] Dean M (2016). “It’s not if I get cancer, it’s when I get cancer”: BRCA-positive patients’ (un)certain health experiences regarding hereditary breast and ovarian cancer risk. Soc Sci Med.

[5] Dean M, Rauscher EA (2017). “It was an Emotional Baby”: previvors’ family planning decision-making styles about hereditary breast and ovarian cancer risk. J Genet Counsel.

[6] Campfield Bonadies D, Moyer A, Matloff ET (2011). What I wish I’d known before surgery: BRCA carriers’ perspectives after bilateral salipingo-oophorectomy. Fam Cancer.

[7] Paluch-Shimon S, Peccatori FA (2018). BRCA 1 and 2 mutation status: the elephant in the room during oncofertility counseling for young breast cancer patients. Ann Oncol.

[8] Chan JL, Johnson LNC, Sammel MD, DiGiovanni L, Voong C, Domchek SM (2017). Reproductive decision-making in women with BRCA1/2 mutations. J Genet Couns.

[9] Daum H, Peretz T, Laufer N (2018). BRCA mutations and reproduction. Fertil Steril.

[10] Donnelly LS, Watson M, Moynihan C, Bancroft E, Evans DGR, Eeles R (2013). Reproductive decision-making in young female carriers of a BRCA mutation. Hum Reprod.

[11] Gietel-Habets JJG, de Die-Smulders CEM, Derks-Smeets IAP, Tibben A, Tjan-Heijnen VCG, van Golde R (2018). Support needs of couples with hereditary breast and ovarian cancer during reproductive decision making. Psycho-Oncol.

[12] Poprzeczny AJ, Stocking K, Showell M, Duffy JMN (2020). Patient decision aids to facilitate shared decision making in obstetrics and gynecology: a systematic review and meta-analysis. Obstet Gynecol.

[13] Ormondroyd E, Donnelly L, Moynihan C, Savona C, Bancroft E, Evans DG (2012). Attitudes to reproductive genetic testing in women who had a positive BRCA test before having children: a qualitative analysis. Eur J Hum Genet.

[14] Rubin LR, Werner-Lin A, Sagi M, Cholst I, Stern R, Lilienthal D (2014). ‘The *BRCA* Clock is Ticking!’: negotiating medical concerns and reproductive goals in preimplantation genetic diagnosis. Hum Fertil.

[15] Stacey D, Légaré F, Lewis K, Barry MJ, Bennett CL, Eden KB, et al. Decision aids for people facing health treatment or screening decisions. Cochrane Consumers and Communication Group, editor. Cochrane Database of Systematic Reviews. 2017; Available from: http://doi.wiley.com/10.1002/14651858.CD001431.pub5. 10.1002/14651858.CD001431.pub5 PMC6478132 28402085

[16] Hoefel L, O’Connor AM, Lewis KB, Boland L, Sikora L, Hu J (2020). 20th anniversary update of the Ottawa decision support framework part I: a systematic review of the decisional needs of people making health or social decisions. Med Decis Mak.

[17] Crabtree BF, Miller WL. Doing qualitative research. Vol. 2nd ed. Thousand Oaks, Calif: SAGE Publications, Inc; 1999. Available from: https://search.ebscohost.com/login.aspx?direct=true&db=nlebk&AN=63285&site=ehost-live.

[18] Jacobsen MJ, O’Connor AM, Stacey D. A workbook for assessing patients’ and practitioners’ decision making needs. 1999. Available from: www.ohri.ca/decisionaid.

[19] Corbin J, Strauss A. Basics of qualitative research, (3rd ed.): techniques and procedures for developing grounded theory. Thousand Oaks: SAGE Publications, Inc.; 2008. 10.4135/9781452230153.

[20] Sandelowski M (2000). Whatever happened to qualitative description?. Res Nurs Health.

[21] Derks-Smeets IAP, Gietel-Habets JJG, Tibben A, Tjan-Heijnen VCG, Meijer-Hoogeveen M, Geraedts JPM (2014). Decision-making on preimplantation genetic diagnosis and prenatal diagnosis: a challenge for couples with hereditary breast and ovarian cancer. Hum Reprod.

[22] Hesse-Biber S (2014). The genetic testing experience of BRCA-positive women: deciding between surveillance and surgery. Qual Health Res.

[23] Kim J, Skrzynia C, Mersereau JE (2015). A pilot study of BRCA mutation carriers’ knowledge about the clinical impact of prophylactic-oophorectomy and views on fertility consultation: a single-center pilot study. J Genet Couns.

