# Supplementary material for: Providers’ perspectives on the reproductive decision-making of BRCA-positive women
Source: BMC Womens Health. 2022 Dec 8;22:506. doi: 10.1186/s12905-022-02093-2 (PMC9730610; doi:10.1186/s12905-022-02093-2)
Supplement: Supplementary file 1 — Additional file 1: This was the semi-structured interview guide used by the authors. [file 12905_2022_2093_MOESM1_ESM.docx]

**Appendix 1**

**Questions for Health Practitioners (BRCA Egg Freezing Needs Assessment)**

1. What decisions do patients with BRCA, prior to risk-reducing salpingo-oophorectomy surgery, make in your practice? Probe: Do they make decisions about electively freezing eggs?

2. Let’s focus on one particular decision: the decision about whether to pursue elective egg freezing prior to risk-reducing salpingo-oophorectomy surgery.

3. What do you see as the main options patients have in making this decision?

4. What do you see as the main advantages/benefits and disadvantages/risks of the options?

| Option | Advantages/Benefits | Disadvantages/risks |
| --- | --- | --- |
| 1. |  |  |
|  |  |  |
|  |  |  |
| 2. |  |  |
|  |  |  |
|  |  |  |
| 3. |  |  |
|  |  |  |
|  |  |  |

| 5. Let’s talk about the difficulty patients have making this decision about egg freezing prior to risk-reducing surgery. How do patients feel when making this decision? |  | Do you think patients feel:   - Unsure about what to do? - Worried what could go wrong - Distressed or upset - Constantly thinking about the decision - Wavering between choices or changing their mind - Delaying the decision - Questioning what is important to them - Feeling physically stressed (tense muscles, racing heartbeat, difficulty sleeping) - other |
| --- | --- | --- |
| 6. What makes the decision difficult for patients? |  | Are patients:   - Lacking information about options, benefits, risks - Lacking information on chances of benefits and harms - Confused from information overload - Unclear about what is important to them - Feeling unsupported in decision making - Feeling pressure from others - Lacking motivation or not feeling ready to make a decision - Lacking the ability or skill to make a decision - Worried about cost |

| 7. What is your usual role in making this decision? | Do you usually:   - Make the decision for the patient - Share the decision with the patient - Provide support or advice for patients to make decision on their own - Other |
| --- | --- |

8. What factors make it difficult for you to support your patients’ decision making?

9. What factors make it easier for you to support your patients’ decision making?

| 10. Who else besides yourself and the patient is usually involved in making this decision? | - Spouse - Family - Friend - Another Health care provider - Other (specify) |
| --- | --- |
| 11. What is their usual role in making the decision (i.e. person mentioned above) | Do they usually:   - Makes the decision for the patient - Share the decision with the patient - Provide support or advice for patients to make the decision on their own - Don’t know - Other (specify) |
| 12. How do patients usually go about making such a decision? | Do they:   - Get information on options - Get information on changes of benefits and risks - Consider personal importance of benefits and risks - Get information on how others go about deciding - Get support from others - Find ways to handle pressure |

13. What would help patients to make this decision?

13A. At what point(s) in the process do you feel decision support should be offered to best help patients with making the decision?

- Prior to, during or after first or subsequent visits to the Familial Ovarian Cancer Clinic? (please specify)
- Prior to, during or after first or subsequent visits to the Fertility Clinic? (please specify)
- Other (specify)
- No help needed

14. What hinders patients (get in the way of) making this decision?

15. Is there anything else that would help overcome barriers in decision making?

16. I will list possible ways to help some people with a decision. Which ones do you think might be useful to your patients for this decision?

| - Counseling from health practitioner | If YES, specify what types |
| --- | --- |
| - Discussion groups of people facing same decision | If YES, specify what type of organization or group |
| - Information materials | If YES, specify content:   - Options - Benefits - Risks - Probabilities of benefits/risks - Help considering personal importance of benefits versus risks - Guidance in the steps of deliberation and communication - Other, specify |
|  | If YES, specify format   - Booklets, pamphlets - Internet – website - App - Videos/DVD - Other, specify |

17. Is there anything else that would help you to do a better job supporting your patients’ decision making?

CHARACTERISTICS OF PRACTITIONER:

18. Number of years in practice

- <5 years
- 5-10 years
- 10-20 years
- Over 20 years

19. Sex (observe)

- Male
- Female

20. Practice discipline and role

Thank you!
