# Supplementary material for: Providers’ perspectives on the reproductive decision-making of BRCA-positive women
Source: BMC Womens Health. 2022 Dec 8;22:506. doi: 10.1186/s12905-022-02093-2 (PMC9730610; doi:10.1186/s12905-022-02093-2)
Supplement: Supplementary file 2 — Additional file 2: This is a patient hand-out that can be used by both non-fertility providers and patients. [file 12905_2022_2093_MOESM2_ESM.docx]

# Frequently Asked Questions for Women* with BReast CAncer (BRCA)1 and BRCA2 Gene Mutations:

**SECTION A: BRCA Implications**

1. **Does having a BRCA gene mutation impact my fertility?**
2. **What decisions should I consider?**

**SECTION B: BRCA and Fertility Options**

1. **Should I see a fertility specialist if I have a BRCA gene mutation? If so, when?**
2. **What options for family building are available to me?**
3. **What is the difference between egg and embryo freezing?**
4. **What does egg or embryo freezing involve?**
5. **What are the pregnancy rates after egg or embryo freezing?**
6. **What does the procedure for an embryo transfer involve?**
7. **What about egg donation?**
8. **What about surrogacy?**

**SECTION C: Preimplantation Genetic Testing (PGT)**

1. **What is PGT and what is the role of testing if I carry the BRCA gene mutation?**

**SECTION D: Costs in Ontario**

1. **It has been recommended that I remove my ovaries to prevent ovarian cancer, are the costs of egg freezing covered by government funding?**
2. **Are the costs of embryo freezing covered by government funding?**
3. **Are the costs of PGT covered by government funding?**
4. **What are the general costs involved in an IVF cycle?**

***** This information refers to “women” as people with internal reproductive organs; however not everyone with internal reproductive organs identifies as a woman and we acknowledge that this information is relevant for anyone assigned female at birth irrespective of their gender identity

**SECTION A: BRCA Implications**

1. **Does having a BRCA gene mutation impact my fertility?**

**Carrying a BRCA gene mutation may have an impact on your pregnancy plans. It may change *when* you decide to have a baby and *how* you have a baby.**

Women are born with a fixed number of eggs. Over time, the eggs decrease in number and quality. Around age 35, the egg number goes down and the chance of these eggs leading to a genetic (chromosomal) abnormality, such as Down syndrome, goes up. By the time a woman is approaching 40 years old, the chance of pregnancy is lower due to these age-related changes. Unfortunately, it is not possible to know how many eggs a specific woman is born with, how many of those eggs are genetically normal, or how quickly that woman will run out of eggs (reach menopause).

While controversial, some women who carry a BRCA mutation may have difficulty having a baby (infertility) or experience earlier menopause (ovarian failure). This may be due to the

BRCA mutation causing a lower egg number. Women have a 50% chance of passing the BRCA mutation on to their children. In addition, women who carry the BRCA mutation are at increased risk of developing breast and/or ovarian cancer and tend to develop these cancers at a younger age. The treatments for breast and/or ovarian cancer, which can include surgery, chemotherapy, or radiation, can also affect egg number and quality.

Risk-reducing surgery (RRS) to prevent ovarian cancer involves the removal of fallopian tubes and/or ovaries. This will impact your ability to have a baby. Your healthcare team will recommend the suggested age for RRS. It is important to note, however, that even if the fallopian tubes and ovaries are removed, a person can still carry a pregnancy if they have a uterus. RRS for breast cancer prevention, which includes removing the breasts, will not impact the ability to have a baby. However, you will not be able to breastfeed.

Treatment or prevention options available to you may impact how and when you decide to start a family. They may lead to you thinking of fertility treatments to increase the chance of pregnancy.

1. **What decisions should I consider?**

You may want to think about the following questions:

1. Do I want to have biological children?
2. Are having children that are genetically related to me very important?
3. How would I feel if I passed on a BRCA mutation to my child?
4. Do I want to carry a pregnancy?

If you have a partner, you may want to discuss these questions together. Consideration of the answers will help your healthcare provider guide you. It is okay to not have all these answers as these are big decisions that require thought and time. We appreciate this can be stressful for people.

**SECTION B: BRCA and Fertility Options**

1. **Should I see a fertility specialist if I have a BRCA gene mutation? If so, when?**

You may want to see a fertility specialist in the following scenarios:

- You are under the age of 35 and have been trying to get pregnant for at least 12 months
- You are over the age of 35 and have been trying to get pregnant for 6 months
- You would like to prevent passing on the BRCA mutation to future children
- You have made the decision to undergo ovarian cancer risk-reducing surgery and would like to pursue egg or embryo freezing before surgery
- You have been diagnosed with breast or ovarian cancer and would like to pursue egg or embryo freezing before treatment

1. **What options for family building are available to me?**

**Depending on your answers to the questions above, you may choose one or a combination of the following options to have a baby.**

| FAMILY BUILDING OPTIONS: | |
| --- | --- |
| Try to have a baby now | - If you are ready to start your family, you can try to conceive now - This may or may not require the assistance of a fertility specialist - Most people will become pregnant within 12 months of trying to conceive |
| Freeze my eggs (before cancer treatment or prevention options) | - If you are not ready to start your family, you may choose to freeze eggs to use when you are ready to have a baby - A pregnancy conceived though your own previously frozen eggs is genetically related to you |
| Freeze embryos through in vitro fertilization (IVF) | - If you are not ready to start your family, you may choose to freeze embryos - Embryos are created before freezing where eggs are fertilized with either a partner’s sperm or with donor sperm - Embryos can undergo preimplantation genetic testing (PGT) to identify whether the embryo carries a BRCA mutation and/or a chromosome abnormality - Embryos can be frozen until you are ready to have a baby - A pregnancy conceived through your own previously frozen embryos is genetically related to you |
| Egg Donation | - If you have low egg number and/or are unable to become pregnant with your own eggs OR you are concerned about passing on the BRCA mutation to your child, you can use donated eggs to have a baby - Donated eggs can be fertilized with either a partner’s sperm or donor sperm - A pregnancy conceived through egg donation is not genetically related to you |
| Surrogacy | - If you are unable or do not want to carry a pregnancy because of the risks to your health, someone else can carry a pregnancy for you - The embryo can be created through egg/embryo freezing with either your own or donated eggs, and with partner or donor sperm - Depending on the eggs used to achieve the pregnancy, you may or may not be genetically related to the child |
| Adoption | - You may choose to adopt a child |
| Child-free living | - You may choose not to pursue pregnancy or having children |

1. **What is the difference between egg and embryo freezing?**

**Egg freezing is also known as “oocyte cryopreservation”.**

**Embryo freezing or “embryo cryopreservation” involves fertilizing (mixing) eggs with sperm prior to freezing (*see diagram directly below*) so that they can be frozen as embryos.**

**
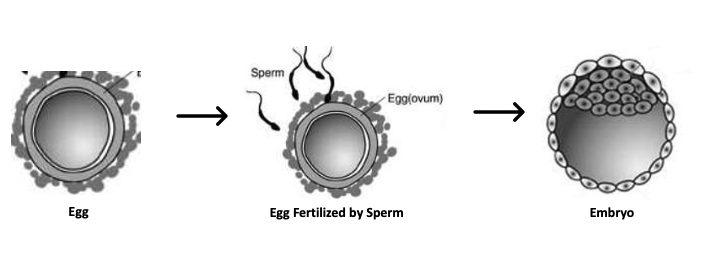
**

Frozen eggs or embryos can remain frozen indefinitely until they are ready to be thawed (unfrozen) for use.

With frozen eggs, all eggs will be thawed when you are ready to have a baby. It is expected that approximately 85% will survive. Those that survive will be injected with sperm. If they fertilize, they will be placed in an incubator to see if any embryos grow. It is important to realize that not all eggs will become an embryo. It generally requires several eggs (4-8 depending on age of the woman) to create a single embryo. If embryos do grow, typically only one embryo will be placed in the uterus at a time. This is called an embryo transfer. Remaining embryos can be frozen again for use at another time. About 95% of good quality embryos will survive the freeze-thaw process.

With frozen embryos, typically only one embryo will be thawed at a time and placed in the uterus.

The choice to freeze embryos or eggs is personal. Many women feel more comfortable with storing unfertilized eggs, especially if they are single. Even women in a relationship may not want to make embryos with a specific partner in case their relationship changes. Recent studies suggest that pregnancy rates are similar when using frozen eggs or frozen embryos.

1. **What does egg or embryo freezing involve?**


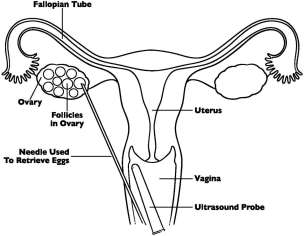
Whether you freeze eggs or embryos, the process to get eggs is the same. The first step is an assessment of the number of eggs in the ovaries (ovarian reserve) which involves an internal (transvaginal) ultrasound and blood test. The second step involves stimulation of the ovaries with self-administered injection medications to produce multiple eggs. Next is an egg retrieval procedure to remove eggs from the ovaries. This procedure will be performed with intravenous medication for sedation and pain management. To retrieve eggs, the doctor will use an ultrasound to see the follicles. They will then place a needle through the top of the vagina into the ovary. All the follicles will be drained. The fluid will be sent to the embryology lab to identify the eggs. The eggs are microscopic and therefore cannot be seen on ultrasound. Shortly after the retrieval you will be told the number of eggs that were retrieved.

1. **What does the procedure for an embryo transfer involve?**


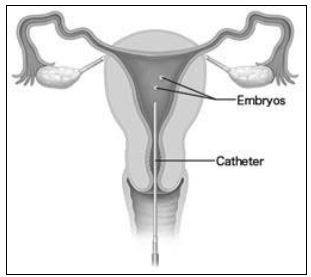
The procedure for placing an embryo into the uterus uses an abdominal ultrasound to see the uterus. The doctor then performs a speculum exam and places a small catheter (tube) into the vagina through the cervix and into the uterus.

A frozen embryo transfer can happen any time after egg retrieval and embryo creation. Most clinics limit transfer to those under the age of 55. You will need to prepare your uterus for implantation with oral and vaginal medication. You will then need transvaginal ultrasound monitoring to determine the best time for an embryo transfer. The embryo will be thawed when your doctor decides that the lining of the uterus on ultrasound is ready for embryo transfer.

1. **What are the pregnancy rates for egg and embryo freezing?**

The chance of having a child from eggs or embryos is related to the age of the woman when the eggs or embryos were first frozen. It is not related to the age of the woman when the embryo is transferred.

Clinic-specific pregnancy rates after egg freezing may be difficult to determine because not all women come back to use their frozen eggs.

In general, if women between the ages of 30-35 are able to freeze 10 mature eggs, their chances of having a baby are about 40%.


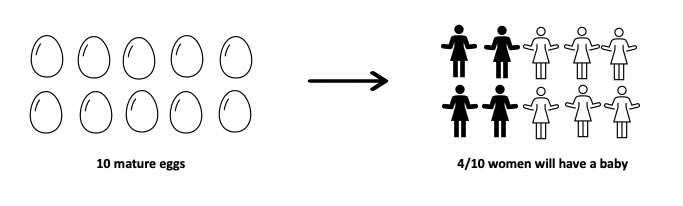


With 20 mature eggs frozen, the chances increase to about 50%.
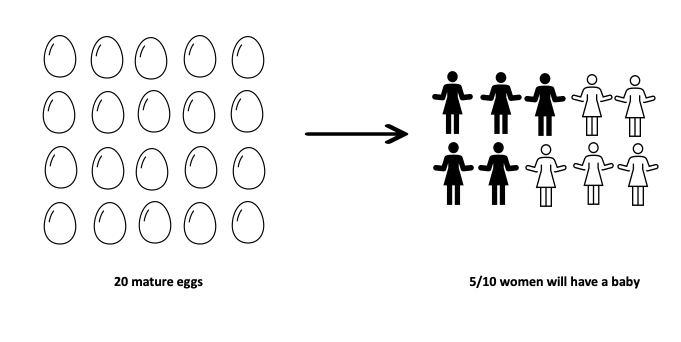


There are many reasons that the chances of having a baby from frozen eggs or embryos are not higher.

- Not all eggs will survive being unfrozen (the thaw)
- Not all eggs that survive the thaw will be able to be fertilized with sperm
- Not all eggs that become fertilized will become an embryo
- Not all embryos are healthy
- Not all embryos will be able to implant in the uterus and lead to a live birth

In addition, as women get older, they will have less eggs and less genetically normal eggs. Older women will need to have more frozen mature eggs to have a higher change of having a baby.

1. **What about egg donation?**

Egg donation is an option for women who:

- Have low egg number or low egg quality
- Have ovarian damage due to surgery or chemotherapy
- Are already in menopause
- Are concerned about passing on the BRCA to their children

An embryo created through egg donation from a frozen egg bank has a high chance of leading to a successful pregnancy. This is because eggs are donated by women between the ages of 21-32.

You may also choose to have a baby using eggs from someone you know who is willing to undergo the process of egg retrieval and egg donation. The chance of having a baby depends on the donor’s age.

1. **What about surrogacy?**

Surrogacy involves having someone else carry a baby for you. This person is known as a surrogate or gestational carrier. An embryo that is created from your own eggs can be placed into a surrogate’s uterus so that they can carry the pregnancy on your behalf.

You may choose to have a surrogate if you have a medical reason limiting you from carrying a pregnancy. Some women who carry the BRCA mutation may choose to use a surrogate because they are currently in treatment for cancer or if their medical team feels it would be the safest option for them.

**SECTION C: Preimplantation Genetic Testing (PGT)**

1. **What is PGT and what is the role of testing if I carry the BRCA gene mutation?**

People who carry a BRCA1 or BRCA2 gene mutation have a 50% chance of passing the gene mutation on to their children. To reduce this risk, preimplantation genetic testing (PGT) can be performed. PGT is an optional test that involves genetic testing on a few cells removed from developed embryos created through IVF. **PGT is performed on embryos only, not eggs.**

PGT for single gene (monogenic) disorders (PGT-M) helps detect genetic mutations like BRCA while PGT to screen for abnormal chromosome number (known as aneuploidy) is known as PGT-A. People can choose either PGT-M or PGT-A or they can combine the two so that their embryos are screened for both BRCA and conditions such as Down Syndrome. PGT is used to help select the best embryo to place in the uterus. PGT cannot change the genetics of an embryo.

The PGT-M lab will design a test unique to your family. You and your family members may be asked to provide a saliva sample to aid in creating this test. This process may take several months. PGT-A, however, does not require a unique familial test.

It is important to remember that neither PGT-M nor PGT-A are treatments for BRCA1 or BRCA2 mutations or abnormal chromosomes. PGT only identifies affected and unaffected embryos to help you and your fertility doctor decide which embryo to place in the uterus.

Written by: E.S. Dason, MD, E.M. Greenblatt MD, E. McMahon, RN, MN, M .Q. Bernardini, MD, A. Cantor, MD, J. Cundamala and C.A. Jones MD
